# Functional Maturation of Human Stem Cell-Derived Neurons in Long-Term Cultures

**DOI:** 10.1371/journal.pone.0169506

**Published:** 2017-01-04

**Authors:** Rebecca S. Lam, Felix M. Töpfer, Phillip G. Wood, Volker Busskamp, Ernst Bamberg

**Affiliations:** 1 Department of Biophysical Chemistry, Max Planck Institute of Biophysics, Frankfurt, Germany; 2 Center for Regenerative Therapies Dresden, TU-Dresden, Germany; University of Pennsylvania, UNITED STATES

## Abstract

Differentiated neurons can be rapidly acquired, within days, by inducing stem cells to express neurogenic transcription factors. We developed a protocol to maintain long-term cultures of human neurons, called iNGNs, which are obtained by inducing Neurogenin-1 and Neurogenin-2 expression in induced pluripotent stem cells. We followed the functional development of iNGNs over months and they showed many hallmark properties for neuronal maturation, including robust electrical and synaptic activity. Using iNGNs expressing a variant of channelrhodopsin-2, called CatCh, we could control iNGN activity with blue light stimulation. In combination with optogenetic tools, iNGNs offer opportunities for studies that require precise spatial and temporal resolution. iNGNs developed spontaneous network activity, and these networks had excitatory glutamatergic synapses, which we characterized with single-cell synaptic recordings. AMPA glutamatergic receptor activity was especially dominant in postsynaptic recordings, whereas NMDA glutamatergic receptor activity was absent from postsynaptic recordings but present in extrasynaptic recordings. Our results on long-term cultures of iNGNs could help in future studies elucidating mechanisms of human synaptogenesis and neurotransmission, along with the ability to scale-up the size of the cultures.

## Introduction

Human neurons derived from stem cells offer a multitude of possibilities, from studying developmental or functional properties of large populations of human neurons, to understanding a specific gene’s effects in a single neuron, to producing cells and tissues for cell therapy and regenerative medicine applications. Acquiring human neurons from precious tissue samples is a slow process that is limited by healthy tissue availability. The ability to repetitively and rapidly produce a large amount of healthy human neurons for *in vitro* studies offers many advantages, and this was not possible before the advent of human embryonic stem cells (ESCs) [1] and induced pluripotent stem cells (iPSCs) in 2006 [2,3]. Typical methods for deriving differentiated and functional human neurons from stem cells require months [4], with multiple steps, and a resulting population of mixed neural cells, including neurons. The resulting cell heterogeneity and batch-to-batch variability make thorough characterization of the differentiated neurons difficult.

Faster methods for obtaining differentiated neurons, by inducible expression of specific neurogenic transcription factors in a single step, cause a rapid transformation of the stem cells into differentiated neurons [5,6]. The induction of Neurogenin-1 and Neurogenin-2 expression in human iPSCs was shown to differentiate neurons, called iNGNs, within only 4 days and produce a nearly pure population of neurons [7]. Induced expression of single transcription factors alone, such as Neurogenin-1 [5], Neurogenin-2 [8,9], ASCL1 [10], and NeuroD1 [11,12], has also been used to obtain differentiated neurons from stem cells.

Although iNGN cells have been extensively studied on the molecular level during neurogenesis and at early postmitotic stages ([7] and ENCODE consortium), we were interested in how iNGN cells functionally mature over time. In this study, we characterized long-term cultures of iNGNs grown in specifically defined conditions. We confirmed that within 14 days iNGNs become electrically active neurons, as previously described with current injection studies [7]. However, it was unclear when and if iNGN cells would become robustly intrinsically functional. Therefore, our aim was to study their functional development at specific time points over the course of months. iNGN cells could survive well beyond 100 days under the specifically defined culture conditions. Patch-clamp electrophysiology recordings were used to assess measures of neuronal function over time, including: cell size, resting membrane potential, voltage-gated channel function, action potential firing properties, spontaneous synaptic activity, postsynaptic and extrasynaptic glutamatergic receptor function. We characterized suitable methods to get iNGNs to express the optogenetic tool channelrhodopsin-2 (ChR2). We also used immunocytochemistry to assess the expression of certain synaptic proteins. In many experiments, we compared data between iNGNs and primary rat hippocampal neurons cultured in the same conditions, to determine the neuronal properties and maturity of iNGNs both functionally and morphologically.

## Materials and Methods

### Human induced pluripotent stem cell culture and neuron differentiation

The modified human induced pluripotent stem cells (iPSCs), called iNGN stem cells, were a kind gift from the lab of Dr. George Church (Harvard Medical School, Boston). The iNGN stem cells are modified PGP1 cells (GM23338, Coriell Institute for Medical Research, Camden, USA) [13], which are iPSCs derived from healthy adult male skin fibroblasts (GM23248, Coriell Institute for Medical Research) through reprogramming using the four Yamanaka transcription factors (Oct-4, Sox2, Klf4, and c-Myc). Lentivirus generation and modifications of PGP1 cells to produce iNGNs were previously published [7], resulting in doxycycline-inducible gene expression of the neuronal transcription factors Neurogenin-1 and Neurogenin-2 in iNGN stem cells. The ENCODE (Encyclopedia of DNA Elements) Consortium [14] has tested iNGN cells according to their standard procedures. The data are freely available (https://www.encodeproject.org, accession number: ENCBS369AAA). The iNGN stem cells were maintained in feeder-free cultures with mTeSR1 medium (Stemcell Technologies, Grenoble, France), without antibiotics, on 6-well plastic plates (Corning Costar, Amsterdam, The Netherlands) coated with Matrigel matrix (Corning). Medium was changed daily or every other day, including gentle phosphate-buffered saline (PBS) (Life Technologies, Darmstadt, Germany) washes, and passaging was done with TrypLE Express (Life Technologies). Cryopreservation of iNGN stem cell stocks was done using mFreSR (Stemcell Technologies). To induce neurogenesis iNGN stem cells were dissociated into single-cell suspension in TrypLE Express, and 6.3x10^4^ cells were plated in neuronal growth medium (NGM = Neurobasal A medium + B27 + GlutaMAX-1; all from Life Technologies), supplemented with 5 μM InSolution Y-27632 (Rho-associated kinase inhibitor (ROCKi); Merck, Darmstadt, Germany) and 0.5 μg/ml doxycycline (Dox; Sigma-Aldrich, Taufkirchen, Germany), onto 14 mm Ø poly-D-lysine-coated (PDL, 100 μg/ml; Merck) glass coverslips (Menzel #1, Thermo Fisher Scientific, Darmstadt, Germany) with rat astrocyte cultures (see below for details). Therefore, the iNGNs were directly differentiated in NGM upon seeding onto coverslips. Viable cell counting was done with the Trypan blue exclusion method using a Luna-FL cell counter (Logos Biosystems, Anyang-si, South Korea). The next day the medium was changed to NGM supplemented with 0.5 μg/ml Dox, and on day 5 the medium was changed to NGM alone, without antibiotics. Half of the NGM was replaced once or twice per week. Neural induction and differentiation of iNGN stem cells was by Dox addition (considered day (d) 0) and the resulting neurons are referred to as ‘iNGNs’ throughout the paper. iNGN stem cells were used between passages 41–52, and our data comprise independent samples of cells that were pooled for analyses, with variability reflected in the error bars.

### ChR2 expression in iNGNs

Adeno-associated virus (AAV) particles were added to iNGNs at both 1 and 5 days after Dox addition, and the cells were assessed (by fluorescent imaging or patch-clamp recordings) from 14–28 days after the first AAV addition (15d – 29d iNGNs). iNGN cultures were transduced with 5x10^8^ genome copies of AAVs in 1.5 ml of media in wells of 24-well plates. The following AAVs were used: AAV2.x-CB7-CI-eGFP-WPRE-rBG, where ‘x’ is either the serotype 1, 5, 8, or 9 (#AV-1589, Penn Vector Core, Philadelphia, USA); AAV2.1-CAG-ChR2-Venus-WPRE-SV40 (#AV-1-20071P, Penn Vector Core), which was previously described [15]; AAV2.2-CAG-ChR2(L132C)-2A-NLS-eGFP-WPRE-bGH (custom-made at Penn Vector Core) differs only in serotype from that previously described [16], and AAV2.1-Synapsin-1-hChR2(L132C)-mKateA (custom-made by B. Roska, FMI Basel; ‘h’ refers to human codon optimization). Successful transduction was monitored by cell fluorescence and/or light-induced currents in patch-clamp experiments.

### Astrocyte cell culture

To aid in iNGN survival [17] and synapse formation [18], astrocytes were co-cultured with iNGNs. Rat cortical astrocytes (Life Technologies) were routinely cultured in astrocyte medium (ACM = DMEM + 15% fetal bovine serum + N2 supplement; Sigma-Aldrich, Sigma,-Aldrich, and Life Technologies, respectively) containing penicillin/streptomycin (100 units penicillin and 100 μg streptomycin/ml final concentration; Sigma-Aldrich), and passaged using StemPro Accutase (Life Technologies). Astrocytes were passaged a minimum of 3 times before co-culture, to ensure there were no neurons, and used until a maximum of passage 12. One to three days before co-culture, 2.5x10^4^ astrocytes were seeded onto coverslips coated with 100 μg/ml PDL, in 24-well plates (Nunc/Thermo Fisher Scientific).

### Rat primary hippocampal neuron culture

Rat procedures complied with German Animal Welfare Legislation as well as the guidelines issued by the Max Planck Society. The institutional animal welfare officer approved the hippocampal preparation from rat pups and all sacrificed animals were reported to the local government (Regierungspräsidium Darmstadt). All rats were maintained at the Max Planck Institute for Brain Research animal facility (Frankfurt, Germany). Maternal Sprague-Dawley rats (Crl:CD(SD), Charles River Laboratories, Sulzfeld, Germany) were received at day 18 after plug observation (timed matings at Charles River Laboratories) and were kept in the animal facility until litter delivery at day 23. The maternal rats were housed under specified-pathogen-free conditions in Type IV Makrolon cages at 20–24°C room temperature, with room lighting set to a 12:12 hour light-dark cycle. Rats received a commercial irradiated diet (ssniff Spezialdiäten, Soest, Germany) and water ad libitum. Cage bedding was a sterilized commercial softwood granulate (Lignocel BK 8–15, J. Rettenmaier & Söhne, Rosenberg, Germany). Dissociated rat hippocampal neurons were prepared from 1-day-old Sprague-Dawley rat pups that were sacrificed, immediately after removal from the mother, by decapitation with sharp scissors. Hippocampi were isolated, pooled, dissociated with papain (Sigma-Aldrich), and 4.0x10^4^ cells were plated onto PDL-coated coverslips in 24-well plates and maintained in NGM. No antibiotics were added to any neuronal (iNGN or rat hippocampal) cultures, since antibiotics negatively affect synaptic activity [19]. For AAV transductions, ChR2-containing AAV particles were added to the media after 5 days *in vitro* (DIV), without the addition of all-trans-retinal. AAV incubation time was between 14–21 days before recordings on 19–26 DIV neurons.

### Electrophysiology

Whole-cell patch-clamp recordings were performed on iNGNs, rat hippocampal neurons, and iNGN stem cells. Artificial cerebrospinal fluid (ACSF) bath solution contained (in mM): 140 NaCl, 1.25 NaH_2_PO_4_·H_2_O, 3 KCl, 1.3 MgSO_4_·7H_2_O, 2 CaCl_2_·2H_2_O, 10 HEPES, pH 7.4 (NaOH). The osmolarity of bath solutions was measured on an osmometer (Semi-Micro Osmometer, KNAUER, Berlin, Germany), and adjusted to 300 mOsm with glucose. NGM was 260 mOsm; however, after switching the neurons from NGM to bath solution, the cells did not undergo osmotic shock and successful recordings could be obtained for at least 45 minutes. Cells were continuously perfused at 1 ml/min with room temperature ACSF. Pipette solution contained (in mM): 135 K-gluconate, 5 KCl, 2 MgCl_2_·6H_2_O, 2 Mg-ATP, 0.2 Na-GTP, 0.1 EGTA, 10 HEPES, pH 7.4 (KOH). Borosilicate pipettes (GB150F-8P, Science Products, Hofheim, Germany) were made on a P-1000 puller (Sutter Instruments, Novato, USA) and fire-polished to measure 4–5 MΩ. Neuron cell bodies were targeted for patch-clamp by visualization with an inverted microscope (Axiovert 40 CFL, Zeiss, Jena, Germany) and 40x lens, and for fluorescent ChR2-expressing neurons the following filter sets were used: XF414 (Omega Optical, Brattleboro, USA) for mKateA and XF105-2 (Omega Optical) for Venus or GFP. Voltage-clamp recordings were acquired with an Axopatch 200A amplifier and Digidata 1322A digitizer, along with Clampex 10.3 software (all from Molecular Devices, Sunnyvale, USA). Cell capacitance values were estimated directly from the software under whole-cell recordings, with values of seal and series resistance always >1 GΩ and <25 MΩ, respectively. Liquid junction potentials were calculated offline in Clampex 10.3 and were 15 mV for the ACSF bath solution and K-gluconate pipette solution and 10 mV for the low-Mg^2+^ ACSF bath solution and Cs-MeSO_4_ pipette solution. Voltage-gated Na^+^ (Na_v_) and K^+^ (K_v_) currents were measured in response to 10 mV, 700 ms steps from a holding potential of -75 mV and a span of -115 to +65 mV. Spontaneous postsynaptic currents (sPSCs) were measured at -75 mV in ACSF, and miniature excitatory postsynaptic currents (mEPSCs) were measured at -75 mV in ACSF containing 1 μM tetrodotoxin (TTX, to block Na_v_ channels) and 100 μM picrotoxin (PTX, to block GABA_A_ receptors). All current recordings were low-pass-filtered at a frequency of 5 kHz and sampled at 20 kHz (50 μs intervals). To determine the contribution of either α-amino-3-hydroxy-5-methyl-4-isoxazolepropionic acid (AMPA)/kainate (KA) receptors, or *N*-Methyl-D-aspartate (NMDA) receptors, 50 μM 6-cyano-7-nitroquinoxaline-2,3-dione (CNQX) or 50 μM (2*R*)-amino-5-phosphonopentanoate (AP5) was applied to the ACSF, to block the respective glutamatergic receptors. To isolate NMDA receptor currents, a low-Mg^2+^ (0.5 mM) ACSF was used, recordings were done at +30 mV, and the pipette solution contained (in mM): 125 Cs-MeSO_4_, 10 CsCl, 2 Mg-ATP, 0.2 Na-GTP, 0.1 EGTA, 10 HEPES, pH 7.4 (CsOH).

For agonist studies, AMPA, KA, and NMDA were bath applied and then washed, and an agonist response was defined as a response where the current returned to baseline levels after the wash step. AMPA and KA recordings were done in ACSF. For NMDA recordings, low-Mg^2+^ ACSF containing 10 μM glycine was used, along with Cs-MeSO_4_ pipette solution.

Current-clamp measurements were low-pass-filtered at a frequency of 5 kHz and sampled at 50 kHz (20 μs intervals). Resting membrane potentials were measured immediately after membrane rupture (whole-cell mode), and input resistance was measured as the steady-state voltage level in response to a negative current injection (between -10 and -100 pA).

Light stimulation of ChR2-expressing neurons was with a 473 nm laser (OptoTech, Wettenberg, Germany) coupled through a 400-μm diameter light fiber attached to a micromanipulator (NMN-21, Narishige, Tokyo, Japan). The light fiber tip was placed 10 μm above the cell body, and before each recording session light power was measured at the fiber tip to determine the light power density. Laser intensity was adjusted with neutral density filters. Laser input to the light fiber was controlled with a shutter driver (VCM-D1, Uniblitz, Rochester, USA) with a 2 mm aperture shutter (LS2, Uniblitz) connected to the digitizer, and light pulses were triggered through the Clampex software.

CNQX and PTX were from Santa Cruz Biotechnology (Dallas, USA), TTX was from Tocris (Bristol, UK), and bath and pipette solution chemicals, NMDA, *S*-AMPA, kainate, and glycine were from Sigma-Aldrich.

### Data analysis

Patch-clamp recordings were analyzed with Clampfit 10.3 and OriginPro 2015G software. Single action potential properties were analyzed from the first AP at the rheobase (the injected current that results in the first AP) for each neuron. Membrane potential was held between -75 to -80 mV. Voltage changes were measured in response to 10 to 20 pA steps of 500 ms current injections, from -40 to +200 pA. Action potentials were defined as spikes that exceeded 0 mV. Amplitude was measured from the baseline to the peak, ‘half-width’ refers to the width at half-maximal amplitude, and thresholds were determined from phase-plane plots (dV/dt vs. V) of the first AP at the rheobase.

To estimate the number of ChR2(L132C) channels in iNGN membranes the following equation was used: N = (current density x specific neuronal membrane capacitance)/(single channel conductance x holding potential x channel open probability). Where N is the number of channels/μm^2^, the specific neuronal membrane capacitance is 0.9 μF/cm^2^ [20], the single channel conductance is 63 fS [16], the holding potential is -75 mV, the open probability is 0.4 [16], and the current density is the measured average photocurrent density.

For spontaneous currents, continuous 4-minute recordings of currents were at -75 mV. The amplitude cutoff value was 5 pA in ACSF containing 1.3 mM Mg^2+^. 10–90% rise times, decay time constants (τ), and charge movement ΔQ (pA·ms) were determined using Clampfit, with decay τ’s determined from a single exponential fitting of the decay portion of the mEPSC. Averaged mEPSCs for each neuron and condition were obtained by aligning the data at 0 mV. Data are reported as averages (means) ± standard error of the mean (SEM). For data sets containing only two groups, statistical comparisons were performed using a paired or unpaired Student’s *t*-test, as appropriate. For data sets containing more than two groups, statistical comparisons were performed using one-way ANOVAs, followed by unpaired two-tailed *t*-tests using a Bonferroni correction factor for multiple comparisons, and P < 0.05 was considered statistically significant.

### Immunocytochemistry

Neurons, grown exactly as for electrophysiology experiments, were fixed with 4% paraformaldehyde in phosphate-buffered saline (PBS) containing Mg^2+^and Ca^2+^ for 20 min, and then incubated for 20 min in 0.1 M glycine and 0.5% Triton X-100 in blocking buffer. Blocking buffer was 4% normal goat serum (Thermo Fisher Scientific #PCN5000) in PBS. Primary antibodies, diluted in blocking buffer, were against: microtubule-associated protein 2 (MAP2, guinea pig polyclonal, 1:1000 dilution, Synaptic Systems #188004, Göttingen, Germany; Antibody Registry AB_1547392), AMPA receptor subunit GluR1 (rabbit polyclonal, 1:200 dilution, Abcam #ab31232, Cambridge, UK; Antibody Registry AB_2113447), AMPA receptor subunit GluR2 (rabbit monoclonal, 1:200 dilution, Abcam #ab133477), and Synapsin-I (rabbit polyclonal, 1:500 dilution, Millipore #AB1543, Antibody Registry AB_1547392). Coverslips were incubated with primary antibodies overnight at 4°C, and then washed 3x with PBS + 0.5% Triton X-100. Secondary antibodies were: goat anti-guinea pig IgG Alexa Fluor 488 (1:1000 dilution, Thermo Fisher Scientific #A11073) and goat anti-rabbit IgG Alexa Fluor 546 (1:1000 dilution, Thermo Fisher Scientific #A11035). The rest of the steps were done in the dark. The coverslips were incubated with secondary antibodies for 30 min at room temperature, and then washed 3x with PBS. Coverslips were counterstained with the nuclear dye 4′,6-diamidino-2-phenylindole (DAPI, 1 μg/ml, Life Technologies) for 10 min, and then washed 3x with PBS. The coverslips were mounted with Aqua-Poly/Mount (Polysciences, Inc.) onto slides and stored in the dark at 4°C until use for confocal microscope imaging.

### Intracellular labeling

iNGNs, grown exactly as for electrophysiology experiments, were filled with Neurobiotin (Vector Laboratories, Burlingame, USA) using a patch-clamp glass pipette containing 0.5% Neurobiotin in the pipette solution, for 20–45 minutes, and 200 ms depolarizing 500 pA current pulses at 1 Hz for 2 min. The pipette was very slowly raised from the cell body and then the coverslip was transferred to a 24-well plate and gently washed in PBS for 5 min before being fixed with 4% paraformaldehyde in PBS containing Mg^2+^and Ca^2+^ for 20 min. Coverslips were washed 3x with PBS and then incubated in Elite ABC reagent (Vector Laboratories) for 2 hr. After washing 3x with PBS, the coverslips were incubated in DAB peroxidase substrate solution (Vector Laboratories) for 10 min, followed by one wash with ddH_2_O. The coverslips were mounted with Aqua-Poly/Mount onto slides and stored upright at room temperature.

### Microscope imaging

Images of Neurobiotin-labeled iNGNs were taken on the same microscope used for patch-clamp recordings using a 10x lens, charge-coupled device camera (uEye UI-2240-C, IDS Imaging Development Systems GmbH, Obersulm, Germany), a 0.5x adapter lens (Zeiss), and uEye 4.4 software. Confocal Z-stack images, compiled from images at 0.5–1 μm intervals, were taken with a 40x water immersion lens on a Zeiss LSM710 microscope using Zen 2009 software. For differential interference contrast (DIC) microscope images, pictures were taken with a 40x lens on a Zeiss Axio Vert.A1 FL-LED microscope using Zen software. Image contrast and brightness were adjusted in Adobe Photoshop CS6.

## Results

### iNGNs in long-term astrocyte co-cultures

The ability to grow neurons in long-term culture is advantageous for studying fundamental questions of neurogenesis and synaptogenesis. Due to long differentiation time lines, typical functional studies on neurons derived from stem cells are done on relatively young neurons. Primary cultures of neurons from rodents have been grown for up to many months [21,22]. Here we applied primary culture techniques to stem cell-derived neurons to obtain healthy long-term cultures (see methods) and studied their function at the single-cell level.

**Fig 1:**
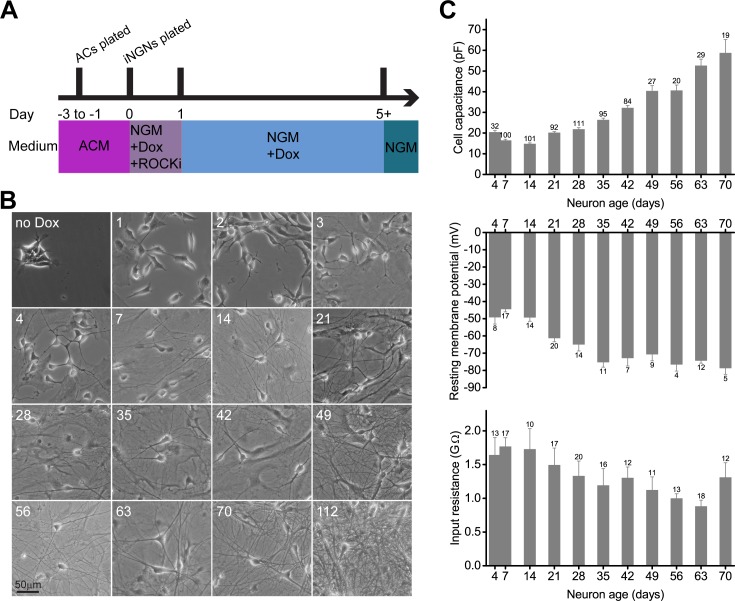
iNGNs mature in long-term culture. (A) Cell culture protocol for deriving long-term cultures of iNGNs. Astrocytes (ACs) were plated onto coverslips between 1 and 3 days before iNGNs. (B) Representative DIC microscope images of iNGNs were taken over the course of long-term co-culture with astrocytes, from 1–112 days. Ages (in days) are labeled in the upper left corners of the images. The uppermost left image shows iNGNs that are undifferentiated (no Dox). (C) Intrinsic properties of iNGNs from 4 to 70 days were measured in whole-cell patch-clamp recordings, and averaged measurements are shown for cell capacitance, resting membrane potential, and input resistance. Error bars denote SEM and numbers next to the errors bars show the number of cells recorded at each age.

In this way, long-term iNGN cultures were feasible for over 100 days. The protocol for conversion of iNGN stem cells into neurons for long-term culture included plating rat astrocytes (ACs) onto coverslips first, adding the stem cells and differentiating them with the addition of doxycycline (Dox), and removing Dox from the cultures at day 5 (Fig 1A). Notably, the cells were directly differentiated in neuronal growth medium that contained no penicillin and streptomycin, which can affect synaptic activity [19]. 25,000 astrocytes and 63,000 iNGNs were seeded onto 14 mm coverslips, and this corresponds to a 1:2.5 ratio of astrocytes:iNGNs. Rat ACs cultured alone, under the same culture conditions as in Fig 1A except no iNGNs were plated, retained their typical flat, spread-out morphology over the course of 56 days *in vitro* (DIV; S1 Fig). Co-cultured iNGN cells with ACs led to longer and more numerous neural dendritic processes and bifurcations with increasing iNGN age in culture (Fig 1B). Intracellular labeling of single neurons with Neurobiotin elucidated the extent of bifurcations in older iNGNs compared to younger iNGNs (S1 Fig). 7d iNGNs had fewer bifurcations than 35d iNGNs (7d iNGNs, 10.8 ± 2.4 branches, n = 4; 35d iNGNs, 18.3 ± 2.0 branches, n = 7; P = 0.02, Student’s *t*-test). If iNGNs were cultured without ACs they had poor viability, and only survived a maximum of 2 or 3 weeks (n = 7 attempts). iNGNs cultured alone had many dendritic processes that showed deterioration over time, with only remnants of iNGN dendrites and cell bodies visible at 28 days (S1 Fig), and the number of viable 28d iNGNs was 0 ± 0 (n = 4 independent samples). In comparison, in 28d and 70d iNGNs grown with astrocytes, the number of viable iNGNs was 18379 ± 2090 (n = 3 independent samples) and 15671 ± 1968 (n = 5 independent samples), respectively (S1 Fig). In whole-cell patch-clamp recordings, capacitance measurements, which are proportional to the area of cell membrane, are an indicator of cellular outgrowth. Comparing the average cell capacitance of age-matched 21d iNGNs, cultured either alone or with ACs, showed that the iNGNs without ACs have a smaller average cell capacitance (21d iNGNs without ACs: 13.5 ± 0.6 pF, n = 4; 21d iNGNs with ACs: 20.2 ± 0.6 pF, n = 92; P = 0.02, Student’s *t*-test). Our data highlight the beneficial effects of astrocyte co-cultures for iNGN long-term viability and cellular outgrowth.

### Passive electrical properties of iNGNs

Whole-cell patch-clamp recordings allowed us to quantitate iNGN neuronal properties at the single-cell level. As well as elucidating function, patch-clamp recordings were used to assess passive electrical properties of iNGNs over the course of time, including cell capacitance, resting membrane potential (RMP), and input resistance (R_i_). Over the course of 70 days, iNGN cultures showed typical signs of neuronal maturation, including an increase in average cell capacitance (Fig 1C, top graph), which corresponds to an increase in cellular outgrowth. This corresponds to the more numerous dendritic processes and bifurcations seen over time (Fig 1B). The average cell capacitance values for 7d, 28d, and 56d iNGNs were 16.4 ± 0.6 pF (n = 100), 21.8 ± 0.8 pF (n = 11), and 40.6 ± 2.6 pF (n = 20), respectively. In comparison, the average cell capacitance values for undifferentiated iNGN stem cells and 13–15 DIV primary rat hippocampal neurons were 15.2 ± 2.9 pF (n = 9) and 32.5 ± 6.6 pF (n = 12), respectively. The average RMP (Fig 1C, middle graph) and R_i_ values (Fig 1C, bottom graph) for iNGNs decreased over 70 days. The average RMP values for 7d, 28d, and 56d iNGNs were -44.4 ± 1.6 mV (n = 17), -64.9 ± 3.8 mV (n = 14), and -76.5 ± 3.9 mV (n = 4), respectively. The average R_i_ values for 7d, 28d, and 56d iNGNs were 1.8 ± 0.1 GΩ (n = 17), 1.3 ± 0.2 GΩ (n = 20), and 1.0 ± 0.1 GΩ (n = 13), respectively. Although the average R_i_ appeared to increase for 70d iNGNs (Fig 1C, bottom graph), the R_i_ of 70d iNGNs was not different compared to any of the other ages (P > 0.05, ANOVA). The larger cell capacitance, more negative RMP, and lower R_i_ over 70 days follow the general trends observed during neuronal maturation [23,24,25]. On the other hand, undifferentiated iNGN stem cells had an average RMP of -42.8 ± 2.7 mV (n = 5) and R_i_ of 3312 ± 786 MΩ (n = 9). These three parameters together are evidence for iNGN maturation over the course of 70 days.

### Active electrical properties of iNGNs and their functional development

Key indicators of a functional neuron are active voltage-gated Na^+^ (Na_v_) and K^+^ (K_v_) channels. Over the 70 days in culture, iNGNs had increases in Na_v_ and K_v_ channel currents, which are key channels responsible for generating action potentials. In response to voltage steps from -95 to +45 mV, Na_v_ channels rapidly open and close resulting in fast inward currents, whereas K_v_ channels are slower to open and result in prolonged outward currents (S2 Fig). Average K_v_ steady-state current densities were measured from the average current over the last 100 ms of the voltage step, and values for 7d, 28d, and 56d iNGNs were 73.6 ± 5.2 pA (n = 24), 104.1 ± 8.8 pA (n = 14), and 78.0 ± 9.7 mV (n = 11), respectively. At older ages, the K_v_ currents showed greater inactivation during the 700 ms voltage steps, as seen in the representative traces from a 56d iNGN (S2 Fig). Therefore, the average K_v_ steady-state current densities are smaller in 56d iNGNs compared to the 28d iNGNs (S2 Fig). Average Na_v_ peak current densities increased with age, and values for 7d, 28d, and 56d iNGNs were -15.2 ± 5.2 pA (n = 20), -55.4 ± 9.1 pA (n = 9), and -45.7 ± 14.9 mV (n = 11), respectively. Na_v_ channel activation curves also had more negative half-activation voltages (V_1/2_) the older the iNGNs were (S2 Fig; 7d iNGNs V_1/2_ = -31.9 ± 1.8 mV, n = 20; 28d iNGNs V_1/2_ = -36.6 ± 1.8 mV, n = 9; 56d iNGNs V_1/2_ = -43.5 ± 1.5 mV, n = 11; between 7d and 56d iNGNs P = 0.0002, ANOVA).

**Fig 2:**
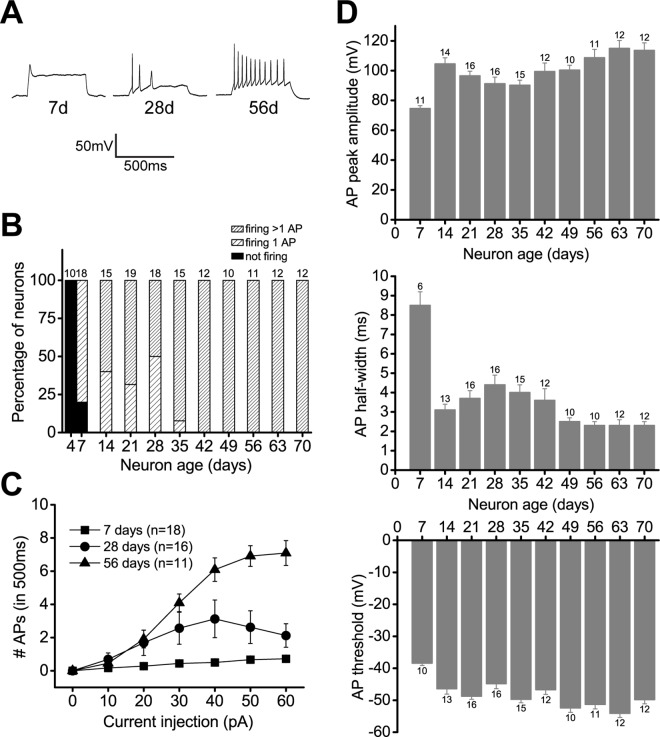
Active properties of iNGNs in long-term culture. (A) Representative current-clamp traces showing action potentials from 7d, 28d, and 56d iNGNs, in response to a 500 ms pulse of current injection from a starting membrane potential of -75 mV. (B) Firing properties of iNGNs from 4 to 70 days in response to current injections of different amplitudes. The numbers of APs were summed over a 500 ms stimulus starting from a membrane potential of -75 mV. Numbers at the top of the histogram bars show the number of cells recorded at each age. (C) Average numbers of APs from 7d, 28d, and 56d iNGNs, in response to current injections up to +60 pA. (D) Action potential properties of iNGNs from 7 to 70 days. Average AP peak amplitudes (top histogram), average AP width at half the maximal peak (half-width, middle histogram), and average AP threshold (bottom histogram) in iNGNs from 7 to 70 days age. Error bars denote SEM and numbers next to the errors bars show the number of cells recorded at each age.

In response to steps of current injections during current-clamp recordings, the fraction of neurons able to fire trains of action potentials (APs) increased over the course of 70 days and by 42 days 100% of neurons could fire trains of APs (Fig 2A and 2B). The maximum number of action potentials a neuron could fire in response to a pulse of 500 ms current injection also increased over the course of 70 days (Fig 2C), and average values for 7d, 28d, and 56d iNGNs were 0.7 ± 0.1 (n = 18), 3.1 ± 1.1 (n = 16), and 7.1 ± 0.7 (n = 11), respectively. The maximum value for one 63d iNGN was 24 APs. APs also had more mature properties over time, including increased peak amplitudes, decreased half-widths, and decreased thresholds (Fig 2D). The average peak amplitude values for 7d, 28d, and 56d iNGNs were 74.6 ± 1.9 mV (n = 11), 91.2 ± 4.5 mV (n = 16), and 108.7 ± 5.5 mV (n = 11), respectively. The average half-width values for 7d, 28d, and 56d iNGNs were 8.5 ± 0.7 ms (n = 6), 4.4 ± 0.5 ms (n = 16), and 2.3 ± 0.2 ms (n = 10), respectively. The average threshold values for 7d, 28d, and 56d iNGNs were -38.4 ± 0.7 mV (n = 10), -44.8 ± 1.6 mV (n = 16), and -51.3 ± 1.4 mV (n = 11), respectively.

Over the course of 70 days, iNGNs showed clear indications of functional maturation, including larger Na_v_ and K_v_ channel currents and increased firing frequencies. These are prerequisite properties for iNGNs to be able to form networks for neural communication.

### Controlling iNGNs with light

Channelrhodopsin (ChR2) is a cation channel from the alga *Chlamydomonas reinhardtii* that is maximally activated by ~470 nm blue light, resulting in cell depolarization [26], and it is one of the most common light-gated ion channels used as a tool to depolarize neurons [27,28]. Creating iNGNs that express ChR2, and other optogenetic tools, is a powerful combination. With the speed of deriving mature human neurons from iNGNs, which can also be functionally manipulated using light, there are numerous possibilities for using ChR2-expressing iNGNs. We used viral transduction to get iNGNs to express ChR2 in order to depolarize the neurons using blue light.

The viral transduction strategy we used was adeno-associated virus (AAV) transduction of the iNGNs. AAV particles were added to cultures of iNGNs at 1 day and again at 5 days after Dox addition (Fig 1A). When AAVs were added to older iNGNs (7d or 14d iNGNs), there were no fluorescent cells observed 2 weeks after transduction (n = 4 independent transductions). To see if there was an AAV serotype more effective for transducing iNGNs, we first used a “serotype kit” with AAVs 2.1, 2.5, 2.8, or 2.9, which all contain GFP. A minimum of 14 days after AAV addition to the cultures, we observed iNGNs for any green fluorescence. All four serotypes (1, 5, 8, and 9) resulted in a portion of fluorescent iNGNs (S3 Fig, n = 5 independent transductions). AAV transduction of rat hippocampal neurons is more efficient, with the majority of the neurons expressing the desired protein. For example, AAV1, AAV8, and AAV9 transduced 80%, 63%, and 43%, respectively, of rat hippocampal neurons [29]. We then used several AAV2 constructs containing ChR2, or the ChR2(L132C) variant of ChR2 (also called CatCh), along with fluorescent proteins, to transduce iNGNs. 15–24 days after AAV addition, patch-clamp recordings were done on fluorescent iNGNs (S3 Fig).

**Fig 3:**
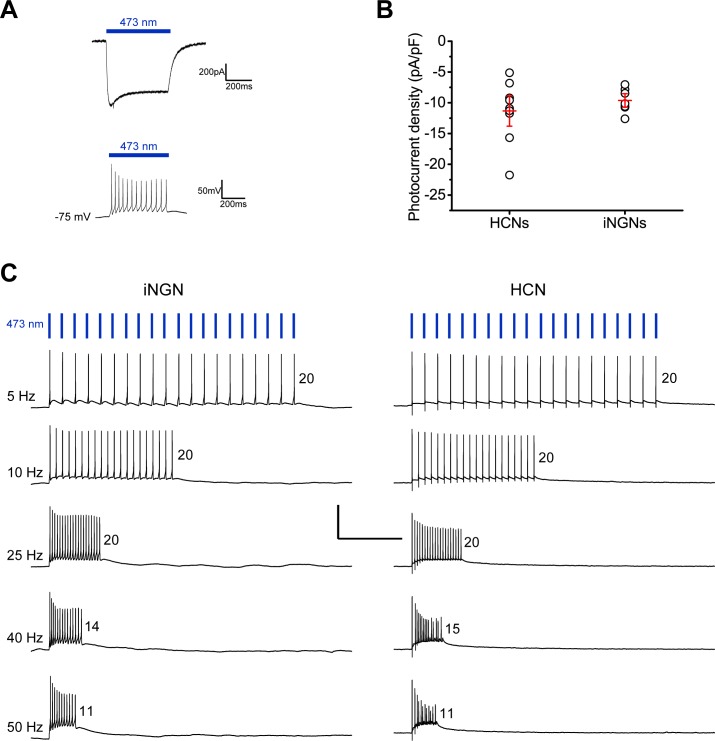
iNGNs expressing ChR2(L132C) can be activated with light. (A) Representative voltage-clamp recording of a 23d iNGN expressing ChR2(L132C)-mKateA (by AAV2 transduction), in response to a 500 ms pulse of 36 mW/mm^2^ 473 nm blue light at a membrane potential of -75 mV (upper trace). Representative current-clamp recording of the same iNGN, in response to a 500 ms pulse of 3.6 mW/mm^2^ 473 nm blue light. Membrane potential was held at -75 mV (lower trace). (B) ChR2(L132C) steady-state current densities, in response to saturating light intensities (20–40 mW/mm^2^), in rat hippocampal neurons (n = 9, 13–26 DIV HCNs) or iNGNs (n = 7, 15d – 24d iNGNs) expressing ChR2(L132C) from AAV2 transductions. One circle represents one neuron, and red crosses show the average steady-state current density as the horizontal line and error bars denote SEM. (C) Representative current-clamp recordings of an iNGN (23d, left traces) and a rat hippocampal neuron (19 DIV HCN, right traces) expressing ChR2(L132C) by AAV transduction. Membrane potential was held at -75 mV and action potentials were driven by different frequencies of 20 pulses (1 ms duration) of 473 nm blue light, denoted by the vertical blue bars (not to scale, and shown only above the 5 Hz stimulation traces for clarity). The blue light power density was 70 mW/mm^2^ for the iNGN and 36 mW/mm^2^ for the HCN. The horizontal and vertical black scale bars denote 1000 ms and 80 mV, respectively. The numbers to the right of each firing trace are the number of action potentials.

AAV2.1-ChR2-Venus expressed poorly in the iNGNs, with <1% expression over the course of numerous attempts (n = 10), and any of the few fluorescent iNGNs appeared unhealthy. A few of the fluorescent neurons that could survive patch-clamp recordings for several minutes at -75 mV, had 473 nm (blue) light-induced currents with a relatively low average steady-state photocurrent density of 2.2 pA/pF (n = 2) at a saturating light power density of 18 mW/mm^2^. The AAV2.1-ChR2(L132C)-mKateA and AAV2.2-ChR2(L132C)-2A-eGFP constructs expressed in a small percentage of iNGNs (~2%). When held at -75 mV, 15d – 24d iNGNs expressing ChR2(L132C) had an average steady-state photocurrent density of 9.6 ± 0.7 pA/pF (n = 7) in response to saturating blue light power densities (20–40 mW/mm^2^) (Fig 3A and 3B). This corresponds to an estimated channel density of ~46 ChR2(L132C) channels/μm^2^. We also compared ChR2(L132C) function between iNGNs and primary cultures of rat hippocampal neurons. In 13–26 DIV rat hippocampal neurons, average steady-state photocurrent density under the same conditions was 11.3 ± 1.6 pA/pF (n = 9) (Fig 3B). Therefore, although AAV2.1-ChR2(L132C) transduced the iNGNs with much lower efficiency, compared to ~80% of rat hippocampal neurons, the cells that did express ChR2(L132C) showed comparable expression levels to rat hippocampal neurons, and average photocurrent densities were not different when compared using a Student’s *t*-test (P = 0.171). In current-clamp recordings, trains of 1 ms pulses of blue light could trigger action potentials with 100% accuracy up to 25 Hz in iNGNs expressing ChR2(L132C) (Fig 3C, left side). In a previous study, similar experiments done in rat hippocampal neurons expressing ChR2(L132C) reliably trigger action potentials up 50 Hz [16] (Fig 3C, right side), which is the maximal firing frequency of these neurons [30]. The maximal firing frequency for light-triggered action potentials is dependent upon the neuron’s innate firing capability. We showed that iNGN average maximal firing frequency in response to long (500 ms) duration current injections (Fig 2A and 2C) increases with age, and a 500 ms duration light stimulation also elucidates an iNGN’s firing capability (Fig 3A).

Using AAV transduction, we could get healthy iNGNs expressing ChR2(L132C), with photocurrent densities on par with those from rat hippocampal neurons expressing ChR2(L132C). The major drawback of AAV transduction in iNGNs was the much lower expression efficiency compared to rat hippocampal neurons.

### Synaptogenesis of iNGNs

#### Presynaptic marker expression

**Fig 4:**
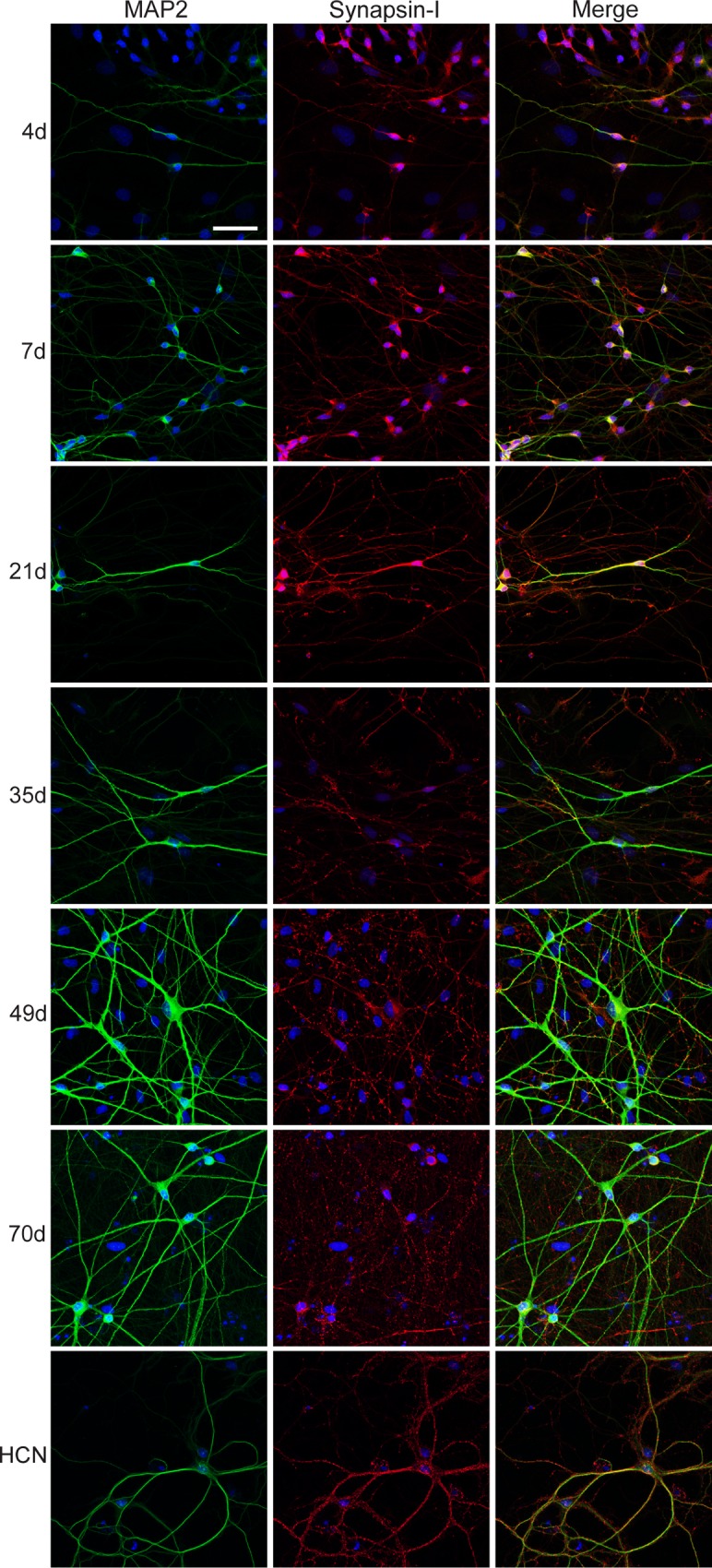
Synapsin-I expression in iNGNs in long-term culture. Representative z-stack confocal microscope images of iNGNs are shown at 4, 7, 21, 35, 49, and 70 days, or of 21 DIV rat hippocampal neurons (HCN). Immunocytochemistry was used to observe MAP2 (green) and Synapsin-I (red) expression and colocalization in iNGNs, along with the nuclear stain DAPI (blue). Synapsin-I is expressed in iNGN dendrites from 4 to 70 days. Ages in days (d) are labeled on the left side of the images, and the white scale bar is 50 μm long.

The ability to form synapses is central to neuronal function. Cellular outgrowth serves to find and form synaptic connections with other cells for neural communication. In order to study the neuronal maturation of iNGNs, we also studied their ability to form synapses using single-cell synaptic recordings and immunocytochemical analyses. The presynaptic vesicular-associated protein Synapsin-I was expressed in iNGNs from 4 to 70 days, and colocalized with the dendritic protein microtubule-associated protein 2 (MAP2) that was also expressed from 4 to 70 days (Fig 4). For comparison, the same antibody staining was done in 21 DIV rat hippocampal neurons (HCN). Synapsin-I had more punctate expression in older iNGNs, and rat HCNs, compared to younger iNGNs. Synapsin-I expression is an indication of synaptic vesicle assembly and tethering to the cytoskeleton, and suggests the priming of iNGNs for synapse formation.

#### Spontaneous network activity

**Fig 5:**
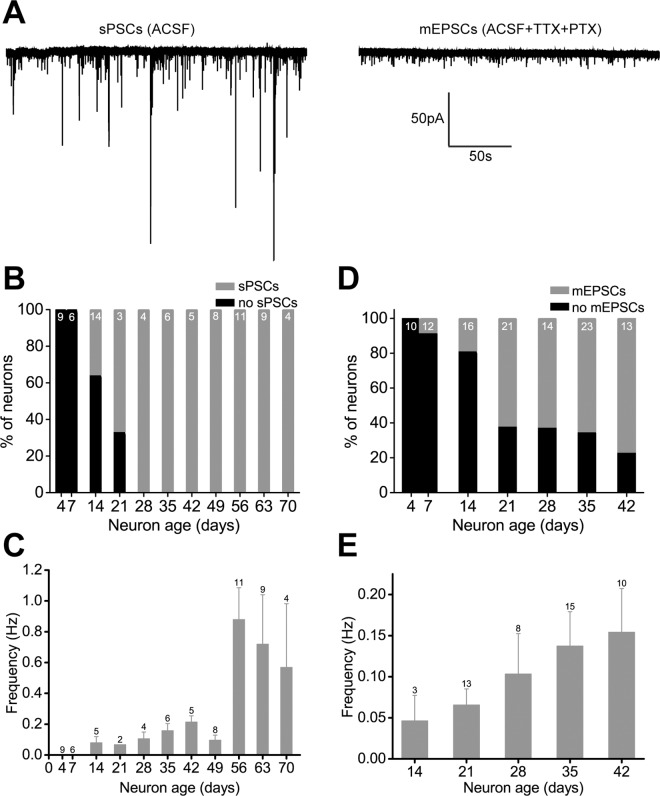
Spontaneous postsynaptic currents (sPSCs) in iNGNs. (A) Representative current traces in a 63d iNGN, showing sPSCs before (left trace) or mEPSCs after (right trace) addition of 1 μM tetrodotoxin (TTX) and 100 μM picrotoxin (PTX) to the ACSF bath solution. Voltage-clamp recordings were done at -75 mV. (B) Fractions of neurons with sPSCs in iNGNs from 4 to 70 days. Numbers at the top of the histogram bars show the number of cells recorded at each age. (C) The average frequency of sPSCs in iNGNs from 4 to 70 days. Error bars denote SEM and numbers next to the errors bars show the number of cells recorded at each age. (D) Fractions of neurons with mEPSCs in iNGNs from 4 to 42 days. Numbers at the top of the histogram bars show the number of cells recorded at each age. (E) The average frequency of mEPSCs in iNGNs from 14 to 42 days. Error bars denote SEM and numbers next to the errors bars show the number of cells recorded at each age.

Functional synaptogenesis in iNGNs was studied in voltage-clamp recordings to observe any spontaneous postsynaptic currents (sPSCs). A representative trace in a 63d iNGN is shown in Fig 5A on the left side, where the downward deflections are sPSCs. After addition of tetrodotoxin (TTX) and picrotoxin (PTX) to the ACSF bath solution, only the AP-independent miniature excitatory postsynaptic currents (mEPSCs) are observed (Fig 5A, right side). Beginning as early as 14 days, approximately 40% of iNGNs had sPSCs (Fig 5B), and by 28 days and older all iNGNs recorded had sPSCs. The average frequency of the sPSCs was ~0.1 Hz between 14 and 49 days, and at 56 days and older was ~10x higher (Fig 5C). Bursts of sPSCs often observed at the older ages were due to AP-induced currents, since they could be blocked with TTX (n = 3), and they had amplitudes of several hundreds of pA in size (Fig 5A). These data indicate that iNGNs in long-term culture have competent network activity as early as 14 days, as well as increased excitability over the course of 70 days.

#### Spontaneous glutamatergic activity

**Fig 6:**
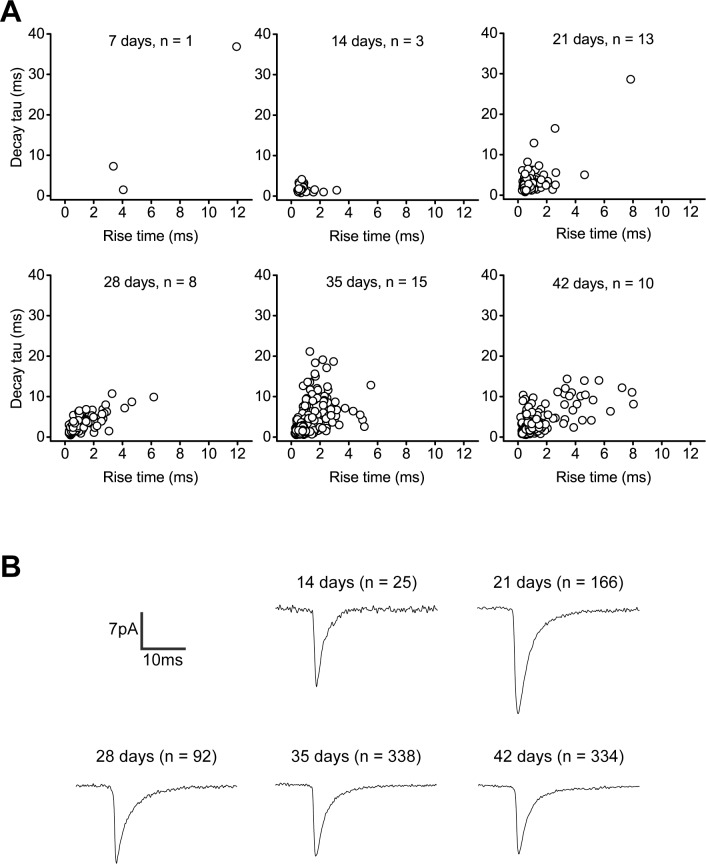
Properties of spontaneous miniature excitatory postsynaptic currents (mEPSCs) in iNGNs. (A) Rise and decay kinetics of mEPSCs in 7d, 14d, 21d, 28d, 35d, and 42d iNGNs. Voltage-clamp recordings were done at -75 mV. Decay τ is plotted against rise time for each age and one circle represents one event. n is the number of cells recorded at each age. (B) Averaged event traces in 14d, 21d, 28d, 35d, and 42d iNGNs. n is the number of events recorded at each age.

Spontaneous vesicle release onto glutamatergic receptors is an important influence on synaptic maturation and homeostasis [31]. Miniature excitatory postsynaptic currents (mEPSCs), which are due to spontaneous presynaptic vesicle release, were recorded at -75 mV and could be recorded as early as 7 days (in one 7d iNGN only, Fig 5D). Between 14, 21, 28, 35, and 42 days, the fraction of iNGNs with mEPSCs went up with increasing age. The average mEPSC frequency showed an increasing trend with increasing iNGN age (Fig 5E), which could be due to release probability changes during this period. The average mEPSC peak amplitudes from 21d iNGNs were larger compared to 28d, 35d, and 42d iNGNs (P = 0.01378, P = 0.00018, P = 0.00006, respectively, ANOVA), but there were no differences between comparisons of any of the other ages (Fig 6A, Table 1). It could be that dendritic electrotonic filtering, where mEPSCs occurring at longer distances from the cell body have smaller measured amplitudes [32], accounts for older iNGN mEPSC amplitudes that are smaller than at 21d. It could also be possible that there are changes in postsynaptic receptor numbers or receptor clustering between iNGNs 21 days and older. The average mEPSC event charge transfers (ΔQ) from 21d iNGNs were larger compared to 14d and 42d iNGNs (P = 0.03393 and P = 0.00003, respectively, ANOVA), and mEPSC ΔQs from 35d iNGNs were larger compared to 42d iNGNs (P = 0.00232, ANOVA, Table 1). Average mEPSC rise times were not different between any of the ages, and decay τ‘s from 35d iNGNs were longer compared to 42d iNGNs (P = 0.02688, ANOVA, Table 1). The rise and decay kinetics of the mEPSCs in 14d, 21d, 28d, 35d, and 42d iNGNs had relatively fast rise time and decay τ values (Fig 6A, Table 1). The kinetics properties suggest the expression of fast postsynaptic AMPA and KA receptors, but not NMDA receptors with much slower kinetics. A small fraction of slower events (rise time >3 ms, decay τ >10 ms) were observed in 21d, 28d, 35d, and 42d iNGNs; however, the majority of events had rise times <3 ms and decay τ’s <10 ms (Fig 6A). Averaged mEPSC traces (all events for each age recorded) are shown in Fig 6B.

**Table 1:** Averaged mEPSC parameters.

	mEPSC parameters				
Parameter	14d (n = 25)	21d (n = 166)	28d (n = 92)	35d (n = 338)	42d (n = 334)
**Peak amplitude (pA)**	17.05 ± 2.02	20.27 ± 0.97	15.95 ± 1.08	15.98 ± 0.43	15.85 ± 0.57
**Rise time (ms)**	0.95 ± 0.12	0.91 ± 0.06	1.26 ± 0.11	1.10 ± 0.04	0.99 ± 0.06
**Decay τ (ms)**	1.93 ± 0.18	3.00 ± 0.21	3.36 ± 0.23	3.54 ± 0.18	2.84 ± 0.14
**ΔQ (pA·ms)**	41.61 ± 5.42	68.60 ± 4.41	58.15 ± 4.56	62.14 ± 2.17	49.97 ± 1.80

Values shown are means ± SEM; d is iNGN age in days; n is the number of events analyzed.

Recordings for mEPSCs were also done at +30 mV and in low-Mg^2+^ ACSF bath solution to optimize conditions for observing any possible larger NMDA receptor-mediated mEPSCs. However, at 21, 28, and 35 days (n = 11), any events had rise times <3 ms and decay τ’s <15 ms, and there were no detectable NMDA receptor-mediated mEPSCs under these optimized recording conditions.

**Fig 7:**
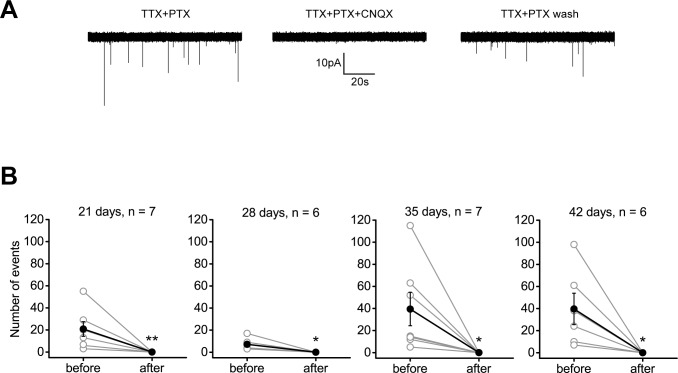
AMPA and KA receptor-mediated mEPSCs in iNGNs. (A) Representative current traces in a 21d iNGN, showing mEPSCs in ACSF bath solution containing 1 μM tetrodotoxin (TTX) + 100 μM picrotoxin (PTX, left trace), 1 μM TTX + 100 μM PTX + 50 μM CNQX (middle trace), and a subsequent wash with 1 μM TTX + 100 μM PTX (right trace). Voltage-clamp recordings were done at -75 mV. (B) Summaries of the number of mEPSC events before or after addition of 50 μM CNQX to 21d, 28d, 35d, and 42d iNGNs. Gray open circles represent the total number of events recorded from one cell and black closed circles are averages of all cells recorded for each age, with error bars denoting SEM and n being the number of cells recorded at each age. The number of events was different before and after addition of CNQX (21d iNGNs: **P = 0.0098, n = 7; 28d iNGNs: *P = 0.0224, n = 6; 35d iNGNs: *P = 0.0403, n = 7; 42d iNGNs *P = 0.0383, n = 6; paired Students *t*-tests).

The AMPA/KA receptor inhibitor CNQX, but not the NMDA receptor inhibitor AP5 (n = 4), inhibited 100% of the mEPSCs in all cells recorded (Fig 7A and 7B). Upon washout of the CNQX from the ACSF bath solution, there was recovery of the mEPSCs. The mean number of events before CNQX addition was not different between the ages (P > 0.3 for all comparisons, ANOVA). The number of events was different before and after (always resulting in 0 events) addition of CNQX at 21, 28, 35, and 42 days (21d iNGNs: P = 0.0098, n = 7; 28d iNGNs: P = 0.0224, n = 6; 35d iNGNs: P = 0.0403, n = 7; 42d iNGNs: P = 0.0383, n = 6; paired Students *t*-tests). This confirmed the presence of postsynaptic AMPA/KA, and lack of NMDA, receptors in 21d, 28d, 35d, and 42d iNGNs.

#### Extrasynaptic glutamate receptors

**Fig 8:**
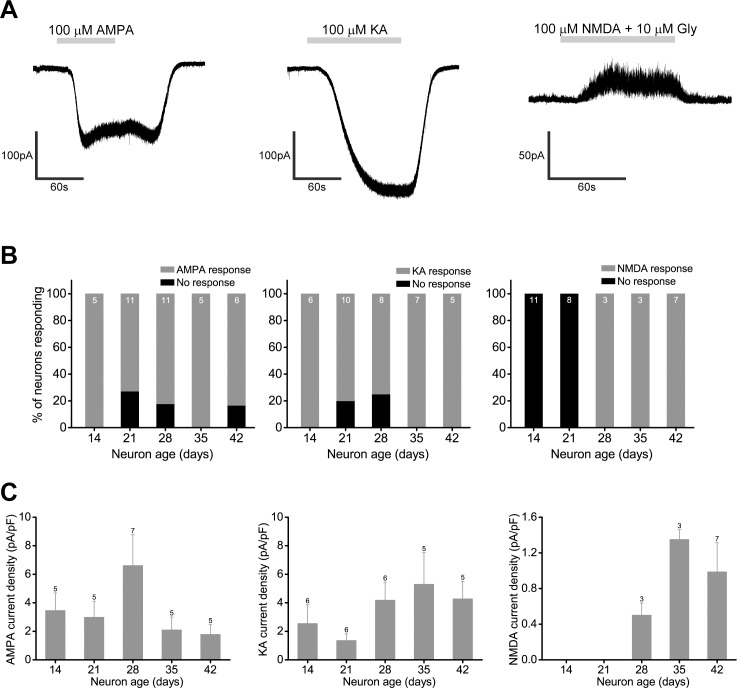
Extrasynaptic AMPA, KA, and NMDA receptor-mediated currents in iNGNs. (A) Representative current traces in three different 42d iNGNs, showing responses to 100 μM AMPA at -75 mV (left trace), 100 μM KA at -75 mV (middle trace), and 100 μM NMDA + 10 μM glycine (Gly) at +30 mV (right trace). (B) Summaries of the percentage of neurons responding to 100 μM AMPA at -75 mV (left histogram), 100 μM KA at -75 mV (middle histogram), and 100 μM NMDA + 10 μM Gly at +30 mV (right histogram). Numbers at the top of the histogram bars show the number of cells recorded at each age. (C) Summaries of the average current densities in response to 100 μM AMPA at -75 mV (left histogram), 100 μM KA at -75 mV (middle histogram), and 100 μM NMDA + 10 μM Gly at +30 mV (right histogram). Error bars denote SEM and numbers next to the errors bars show the number of neurons recorded at each age.

To look at extrasynaptic glutamate receptor expression and function in iNGNs, glutamate receptor agonists were bath-applied during voltage-clamp recordings. 100 μM *S*-AMPA and 100 μM KA induced large inward currents at -75 mV (Fig 8A, left and middle traces). 100 μM NMDA, along with its co-agonist 10 μM glycine (Gly), induced smaller, but detectable, outward currents at +30 mV (Fig 8A, right trace). Most of the iNGNs responded to both exogenous AMPA and KA at 14, 21, 28, 35, and 42 days (Fig 8B, left and middle histograms), and average current densities were ~2–4 pA/pF for both AMPA and KA (Fig 8C, left and middle histograms). At 7 days (n = 4), 14 days (n = 11), and 21 days (n = 8), none of the cells responded to exogenous NMDA (Fig 8B, right histogram). At 28 days, 35 days, and 42 days (Fig 8B and 8C, right histograms), iNGNs responded to exogenous NMDA; however, the average current densities were less than those for exogenous AMPA or KA, ~0.5–1 pA/pF. This suggests that NMDA receptors are expressed in iNGNs, but are less abundant than AMPA and KA receptors, and are functional by 28 days and older, but not at 14 days and 21 days. Together with the mEPSC recordings, the data show that 28d, 35d, and 42d iNGNs have NMDA receptors extrasynaptically, but receptor activity is not detectable postsynaptically.

#### AMPA receptor expression

While iNGNs show functional synapses at early ages, their synapses dominantly express AMPA and KA, but not NMDA, receptors from 14 days– 42 days, as suggested from the previous mEPSC and extrasynaptic receptor analysis. AMPA receptors are tetramers made up of four subunits (GluR1–4). GluR1 and GluR2 receptor expression was assessed in iNGNs over the course of 70 days using immunocytochemistry. 21 DIV rat hippocampal neurons were also immuno-stained under identical conditions. GluR1 dendritic expression was clearly seen at older ages, when it was colocalized with MAP2 (S4 Fig). GluR2 expression could also be seen in iNGN dendrites (S5 Fig). However, at 7 days and 14 days, GluR2 expression was dominant in the cell body.

## Discussion

Most studies showing the functionality of neurons derived from stem cells are performed on a shorter-term time scale, up to a couple of weeks. Inducible expression of neurogenic transcription factors affords a kind of *in vitro* neuronal “birth date” for iNGNs, not possible in other neuronal differentiation protocols for stem cells. Here, we systematically studied iNGN function at specific time points, up to months in culture after the induction of neurogenesis.

Cultures of pure differentiated neurons derived from stem cells, such as by using induced expression of neurogenic transcription factors, fail to produce healthy, long-living, and functional neurons and neuronal networks. When iNGNs were grown alone in neuronal medium, they thrived poorly at 21 days and did not survive past 28 days. Most culturing conditions for pure stem cell-derived neurons have been based on conditions for primary rodent, primate, and human neurons, and these conditions mostly underperform with stem cell-derived neurons. Different strategies to enable long-term cultures (~100 days) of stem cell or iPSC-derived neurons have been used, such as growing the neurons on micropatterned surfaces [33] and astrocyte co-culture [34]. It has also been shown that adding mouse neurons in addition to astrocytes significantly increases the maturity of induced stem cell-derived neurons [9]. We successfully grew iNGNs in long-term cultures using astrocyte co-cultures and a simple defined protocol that results in functional neuronal networks. The astrocytes could be helping to “micropattern” the single iNGNs in our studies, since we observed no cell clustering, that hinders long-term cultures [33], and in this way act as physical support for the neurons. The astrocytes also exert their support through secreted factors that benefit neuron viability and synaptogenesis [17,18].

Long-term culture studies of other induced-neurons that could be useful to directly compare our functional data with are not yet available; however, iNGNs showed many hallmark properties for functionally mature neurons. At the younger ages, 21 days or less, we could compare iNGNs with similarly aged ASCL1-induced neurons, where both neuron populations had similar values for resting membrane potential (RMP), cell capacitance, input resistance (R_i_), and action potential (AP) threshold; however, AP amplitude was larger in iNGNs than in ASCL1-induced neurons [10]. The passive electrical properties and bifurcations of iNGNs further matured beyond 21 days, and cell capacitance increased to ~50 pF by 7 weeks, indicating neurite outgrowth.

iNGNs develop a mature phenotype more quickly than neurons derived from stem cells through multi-step differentiation protocols. 70d iNGNs had a RMP of ~-79 mV and R_i_ of ~1.3 GΩ (Fig 1C). In comparison, in two studies of human neurons derived from iPSCs at week 7 and 10 of differentiation, RMP was ~-28 mV and R_i_ as ~2.7 GΩ [35] and RMP was ~-49 mV and R_i_ was ~1.7 GΩ [36], respectively. Similar to young developing rodent neurons [25], iNGN RMP was depolarized (~-45 mV) at 7 days and steadily decreased to ~-75 mV by 35 days. Undifferentiated iNGN stem cells had a high R_i_, whereas over the course of long-term culture iNGN neurons had a decreasing R_i_. One contribution to the decrease in R_i_ is the corresponding increase in Na_v_ and K_v_ channel expression.

During the first 4 days of iNGN differentiation, Busskamp et al. showed an increase in gene expression of many of the Na_v_ and K_v_ channel subtypes [7]. We observed larger Na_v_ and K_v_ channel current densities with increasing ages (S2 Fig). By 42 days in culture, 100% of all 57 recorded iNGNs could fire trains of APs and older iNGNs could fire at higher firing frequencies (Fig 2B and 2C), up to 48 Hz. This is similar to cultures of rat excitatory pyramidal hippocampal neurons, which have maximal firing frequencies of 40–50 Hz [30]. AP properties matured with increasing iNGN age, such as decreasing half-width, and this facilitates iNGNs to fire at higher frequencies. iNGN AP properties matured more quickly compared to neurons derived from stem cells through multi-step differentiation protocols. 70d iNGNs had APs with a peak amplitude of ~114 mV and half-width of ~2.3 ms (Fig 2D). In comparison, in one study of human neurons derived from iPSCs at week 7 of differentiation, AP peak amplitude was ~39 mV and half-width was ~3.2 ms [36].

Upon blue light stimulation, the active light-gated cation channel ChR2 results in cell depolarization. By using AAVs, we could get a fraction of iNGN cells expressing the ChR2 variant ChR2(L132C) [16] and thereby control iNGN activity with blue light stimulation. Although the AAV transduction efficiency was 10x less in iNGNs than in rat hippocampal neurons, the iNGNs that expressed ChR2(L132C) had similar levels of channel expression (Fig 3B). Whether to manipulate a single neuron or whole neuronal networks of iNGNs, ChR2-expressing iNGNs allow the option of controlling iNGN activity with high temporal and spatial precision. In the future, it will be important to develop refined methods where a majority, rather than a minority, of iNGNs express ChR2. Two key improvements would be higher ChR2 expression efficiency and stable ChR2 expression over longer time courses.

Neurons expressing ChR2 offer a viable therapeutic approach for some diseases, such as for restoring visual functions in blind retinas, for example, by expressing ChR2 in ganglion cells [37] or ON bipolar retinal cells [38,39]. *In vivo* experiments have been done using mouse ESCs expressing ChR2, which were then transplanted and integrated as motor neurons to restore muscle function in denervated muscles in mice [40]. In the future, iNGNs robustly expressing ChR2 could have applications in translational medicine.

Human synaptogenesis is difficult to study from healthy human neural tissue samples, and stem cell-derived neurons offer an easier way gain insight into the development of human synapses. As seen by immunohistochemistry, iNGNs had clear Synapsin-I protein expression as early as 4 days (Fig 4), which was previously seen [7]. Correspondingly, we could detect synaptic activity in ~25% of 14d iNGNs (Fig 5), which shows they had the necessary pre- and postsynaptic architecture for functional synapses. By 21 days and older, the majority of iNGNs had functional synapses. We also observed large (hundreds of pA) spontaneous postsynaptic currents in older iNGNs, which were AP-dependent and indicate increased iNGN excitability over time. Our studies on the synaptic properties of iNGNs are not easily comparable to other studies since our culture conditions included only the iNGNs with astrocytes. For example, the ASCL1-neurons had synaptic activity but when cultured together with primary hippocampal neuron cultures [10]. Here we show synaptic activity with iNGNs cultured only with astrocytes, and without an additional neuronal cell type [9,41].

Spontaneous release onto glutamatergic receptors plays a role in synaptic homeostasis [31] and in the regulation of dendritic protein synthesis [42]. Furthermore, spontaneous glutamate release acting on AMPA receptors is important for the maintenance of dendritic spines [43]. Miniature EPSC recordings, measuring postsynaptic currents in response to spontaneous vesicle release, showed a trend for increasing event frequency with age. This could indicate that the number of iNGN synapses increases from 14–42 days and/or there is an increase in vesicle release probability.

During the first 4 days of iNGN differentiation, Busskamp et al. showed there was an increase in gene expression of glutamatergic receptors [7]. In our studies, AMPA/KA receptors were present and post-synaptically functional as evident from mEPSC recordings. The mEPSCs had fast kinetics, indicative of AMPA/KA receptors, as opposed to the much slower kinetics of NMDA receptors. An AMPA/KA receptor inhibitor blocked 100% of the mEPSCs (Fig 7). NMDA receptor-mediated mEPSCs were not detectable, even in conditions which remove the extracellular Mg^2+^ block according to NMDA receptor IV plots [44]. Extra-synaptic glutamatergic receptor stimulation, using AMPA, KA, or NMDA, showed sizable responses to AMPA and KA, but smaller responses to NMDA. NMDA currents also had a later onset compared to AMPA and KA currents (Fig 8).

AMPA receptors mediate fast excitatory glutamatergic neurotransmission in the mammalian central nervous system. They are tetramers, often heterotetramers, made up of four subunits, called GluR1–4 or GluA1–4 [45]. GluR1 and GluR2 are vital in the normal functioning of the human brain, and also have roles in neurological diseases, such as Alzheimer’s disease [46,47] and drug addiction [48,49]. Evidence for AMPA receptor expression in iNGN synapses was confirmed by the kinetic properties of mEPSCs (Fig 6 and Table 1), inhibitor studies (Fig 7), and by immunocytochemistry using antibodies against GluR1 and GluR2 (S4 Fig and S5 Fig). These long-term cultures of iNGNs could be a convenient model to study neurological disorders where GluR1 and GluR2 are implicated. They could also be used for drug screening on specific neural targets, such as the GluR2-containing AMPA receptors.

Human neurons derived from iPSCs are an important tool for convenient brain disease modelling. Disease-specific neurons can be made and studied *in vitro*, which is an advantage to learn about neuronal and network properties in pathological conditions. Neurons differentiated from iPSCs have been derived from patients with neurodegenerative or neurodevelopmental disorders, such as spinal muscular atrophy [50], Down’s syndrome [51,52], familial dysautonomia [53], amyotrophic lateral sclerosis [54,55], and Alzheimer’s disease [56,57].

Alternatively, disease model neurons can be created *in vitro* using genome-editing strategies on healthy stem cells, which in turn can be differentiated into neurons. Fattahi et al. used the CRISPR/Cas9 system [58] on human ESCs to target the EDNRB locus to model Hirschsprung disease in the resulting enteric nervous system cells. They also used optogenetics (ChR2) and light stimulation of the enteric neurons to measure smooth muscle cell contractile responses [59]. A model for epileptic encephalopathy was made using gene-targeted ESCs, also in combination with optogenetics (ChR2/ChiEF) [60]. Together with the above-mentioned methods, modified iNGNs could also be created for disease model studies, along with ChR2 as a tool to precisely activate the iNGNs.

iNGN cultures can also offer a more thorough system to characterize and compare new optogenetic tools. The rodent cortical or hippocampal neuron cultures commonly used to characterize light-gated channel or pump function can complicate conclusions due to the variable neuronal properties, from numerous neuron subtypes, in one culture. These primary cultures typically contain approximately 15% interneurons [61,62], and ChR2 function has variable effects on different neuron subtypes [63]. On the other hand, iNGN cultures offer a more homogenous population of neurons and the ability to control many conditions, such as the age of the neurons.

In summary, we have generated long-term cultures of functional iNGN neuronal networks that show many of the hallmarks of neuronal maturation and form glutamatergic synapses rich in postsynaptic AMPA, but not NMDA, receptors. We also generated iNGNs expressing ChR2, which enable opportunities for manipulating iNGN activity in future studies. iNGNs cultures offer a greater degree of control to study mechanisms of early human synapse formation, and we have the ability to answer new questions about connectivity. Further studies can precisely define iNGN synapse architecture at the molecular level, and the ability to scale-up cultures could produce large iNGN networks for high-throughput studies on neural networks.

## Supporting Information

S1 Fig iNGNs and rat astrocytes grown alone. (A) Representative DIC microscope images of iNGNs grown alone were taken at 4, 7, 14, 21, and 28 days after addition of doxycycline. Ages (days) are labeled in the upper left corners of the images. (B) The average numbers of viable iNGNs were counted from DIC microscope images of 28d iNGNs grown alone, 28d iNGNs grown with astrocytes, and 70d iNGNs grown with astrocytes. Error bars denote SEM and numbers next to the error bars show the number of independent samples. (C) Representative DIC microscope images of 7, 14, 21, and 56 DIV rat astrocytes grown alone. Ages (days *in vitro*) are labeled in the upper left corners of the images. (C) Representative images from Neurobiotin-labeled 7d and 35d iNGNs. Only a portion of the 35d iNGN is shown.
(TIF)

S2 Fig Voltage-gated currents in iNGNs in long-term culture. (A) Representative voltage-gated currents from four cells: 7d, 28d, and 56d iNGNs. Currents are shown in response to 700 ms voltage steps from -95 mV to +45 mV, in +10 mV increments, from a holding potential of -75 mV. Ages are labeled below the current traces. Zoomed-in traces below show the fast Na_v_ peak currents (they open and close within several milliseconds) at -25 mV. The rightmost trace shows currents after addition of the Na_v_ inhibitor TTX (1 μM). (B) Average steady-state K_v_ current densities from 7d, 28d, and 56d iNGNs. (C) Average peak Na_v_ current densities in 7d, 28d, and 56d iNGNs. (D) Average Na_v_ channel activation in 7d, 28d, and 56d iNGNs. Na^+^ conductance (G) was determined from G = I/(V-V_Rev_), where I is the measured peak current at a specific voltage (V), and V_Rev_ is the extrapolated reversal potential. G was normalized to G_max_, the maximum recorded conductance for each cell. The lines are curves showing averaged Boltzmann fits to individual recordings. Mean values are: 7d iNGNs V_1/2_ = -30.5 ± 1.5 mV, n = 20; 28d iNGNs V_1/2_ = -35.7 ± 1.6 mV, n = 9; 56d iNGNs V_1/2_ = -44.3 ± 1.2 mV, n = 11; ANOVA showed that V_1/2_ values differed between 7d and 56d iNGNs, P = 0.0002. Error bars show SEM and n is the number of neurons recorded.
(TIF)

S3 Fig AAV transductions of iNGNs. Representative pairs of microscope images of iNGNs show bright-field images on the left side and fluorescent images on the right side. (A) GFP expression using four different AAV serotypes. Images were taken from 15d iNGNs, 14 days after addition of the AAVs. (B) Two different ChR2(L132C) constructs expressed in iNGNs. The images on the left were taken from 29d iNGNs and show mKateA fluorescence 28 days after addition of AAV2.1-Synapsin-1-hChR2(L132C)-mKateA. The images on the right were taken from 15d iNGNs and show GFP fluorescence 14 days after addition of AAV2.2-CAG-ChR2(L132C)-2A-NLS-eGFP-WPRE-bGH.
(TIF)

S4 Fig AMPA receptor GluR1 subunit expression in iNGNs in long-term culture. Representative z-stack confocal microscope images of 4d, 14d, 28d, 35d, 42d, and 70d iNGNs, or of 21 DIV rat hippocampal neurons (HCN). Immunocytochemistry was used to observe MAP2 (green) and GluR1 (red) expression and colocalization in iNGNs, along with the nuclear stain DAPI (blue). Ages in days (d) are labeled on the left side of the images, and the white scale bar is 50 μm long.
(TIF)

S5 Fig AMPA receptor GluR2 subunit expression in iNGNs in long-term culture. Representative z-stack confocal microscope images of 4d, 7d, 14d, 35d, 49d, and 70d iNGNs, or of 21 DIV rat hippocampal neurons (HCN). Immunocytochemistry was used to observe MAP2 (green) and GluR2 (red) expression and colocalization in iNGNs, along with the nuclear stain DAPI (blue). Ages in days (d) are labeled on the left side of the images, and the white scale bar is 50 μm long.
(TIF)
