# Supplementary figures and images for: Functional Maturation of Human Stem Cell-Derived Neurons in Long-Term Cultures
Source: PLoS One. 2017 Jan 4;12(1):e0169506. doi: 10.1371/journal.pone.0169506 (PMC5215418; doi:10.1371/journal.pone.0169506)

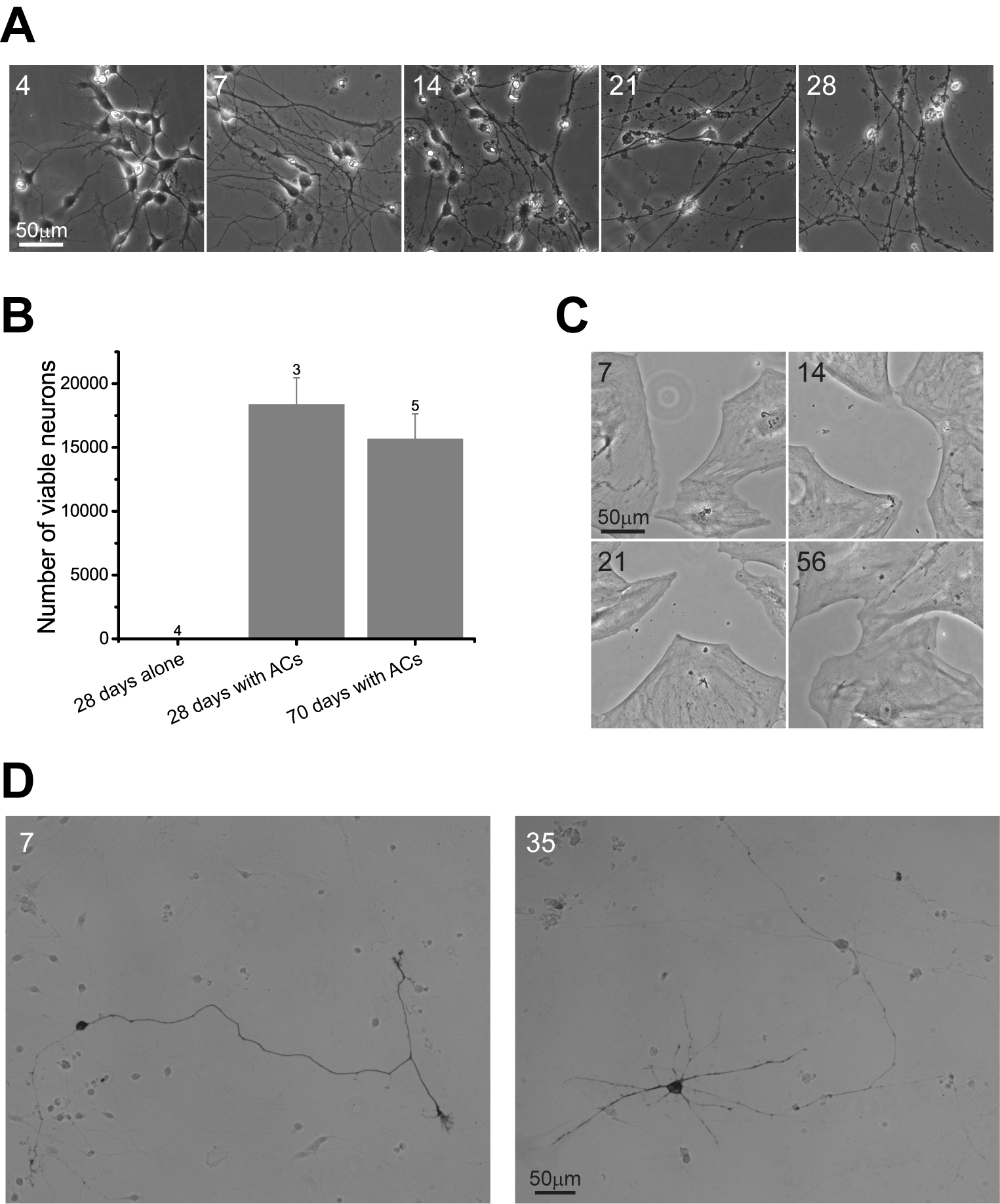

Supplement: S1 Fig — (A) Representative DIC microscope images of iNGNs grown alone were taken at 4, 7, 14, 21, and 28 days after addition of doxycycline. Ages (days) are labeled in the upper left corners of the images. (B) The average numbers of viable iNGNs were counted from DIC microscope images of 28d iNGNs grown alone, 28d iNGNs grown with astrocytes, and 70d iNGNs grown with astrocytes. Error bars denote SEM and numbers next to the error bars show the number of independent samples. (C) Representative DIC microscope images of 7, 14, 21, and 56 DIV rat astrocytes grown alone. Ages (days in vitro) are labeled in the upper left corners of the images. (C) Representative images from Neurobiotin-labeled 7d and 35d iNGNs. Only a portion of the 35d iNGN is shown. (TIF) [file pone.0169506.s001.tif]

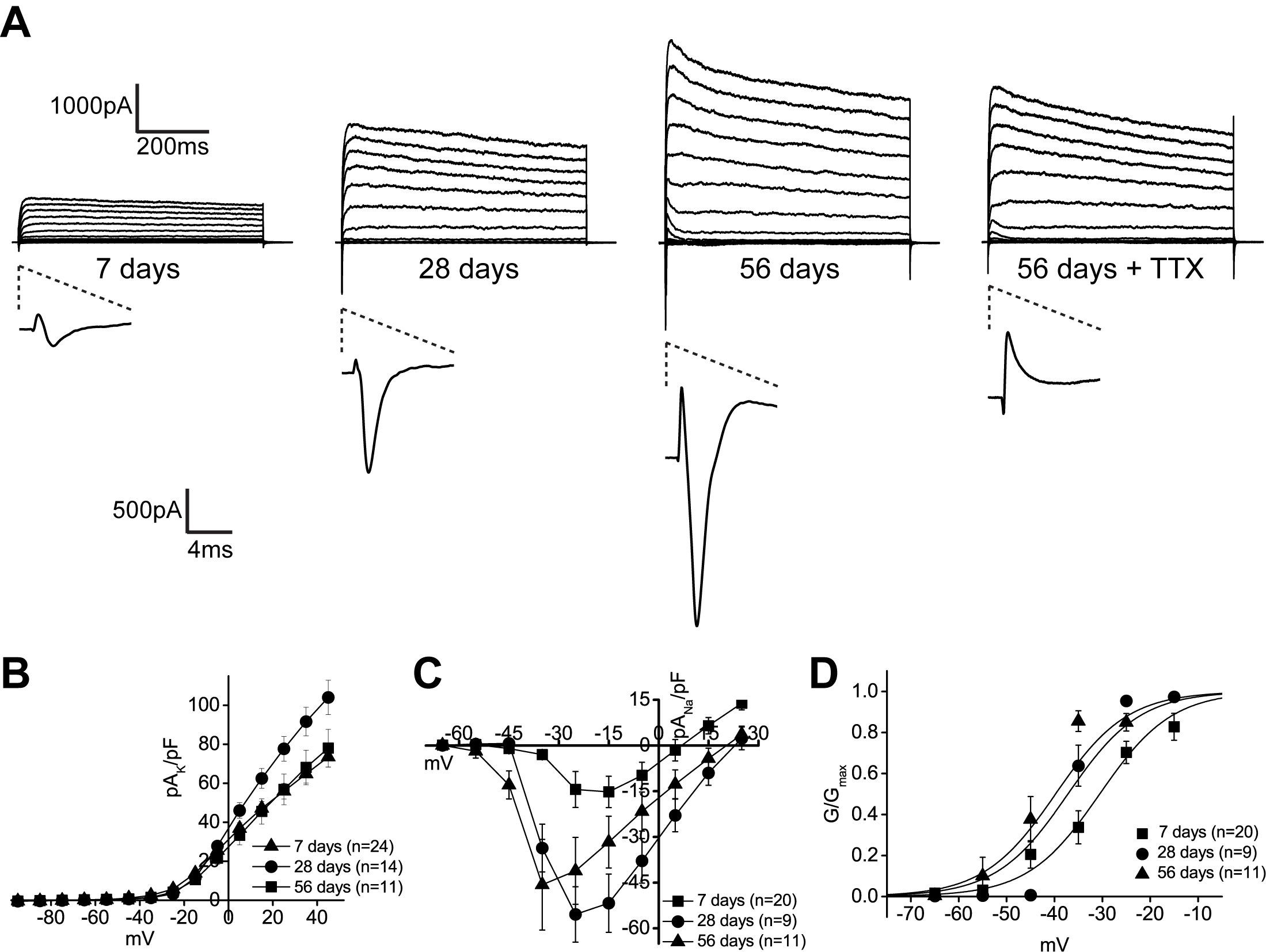

Supplement: S2 Fig — (A) Representative voltage-gated currents from four cells: 7d, 28d, and 56d iNGNs. Currents are shown in response to 700 ms voltage steps from -95 mV to +45 mV, in +10 mV increments, from a holding potential of -75 mV. Ages are labeled below the current traces. Zoomed-in traces below show the fast Nav peak currents (they open and close within several milliseconds) at -25 mV. The rightmost trace shows currents after addition of the Nav inhibitor TTX (1 μM). (B) Average steady-state Kv current densities from 7d, 28d, and 56d iNGNs. (C) Average peak Nav current densities in 7d, 28d, and 56d iNGNs. (D) Average Nav channel activation in 7d, 28d, and 56d iNGNs. Na+ conductance (G) was determined from G = I/(V-VRev), where I is the measured peak current at a specific voltage (V), and VRev is the extrapolated reversal potential. G was normalized to Gmax, the maximum recorded conductance for each cell. The lines are curves showing averaged Boltzmann fits to individual recordings. Mean values are: 7d iNGNs V1/2 = -30.5 ± 1.5 mV, n = 20; 28d iNGNs V1/2 = -35.7 ± 1.6 mV, n = 9; 56d iNGNs V1/2 = -44.3 ± 1.2 mV, n = 11; ANOVA showed that V1/2 values differed between 7d and 56d iNGNs, P = 0.0002. Error bars show SEM and n is the number of neurons recorded. (TIF) [file pone.0169506.s002.tif]

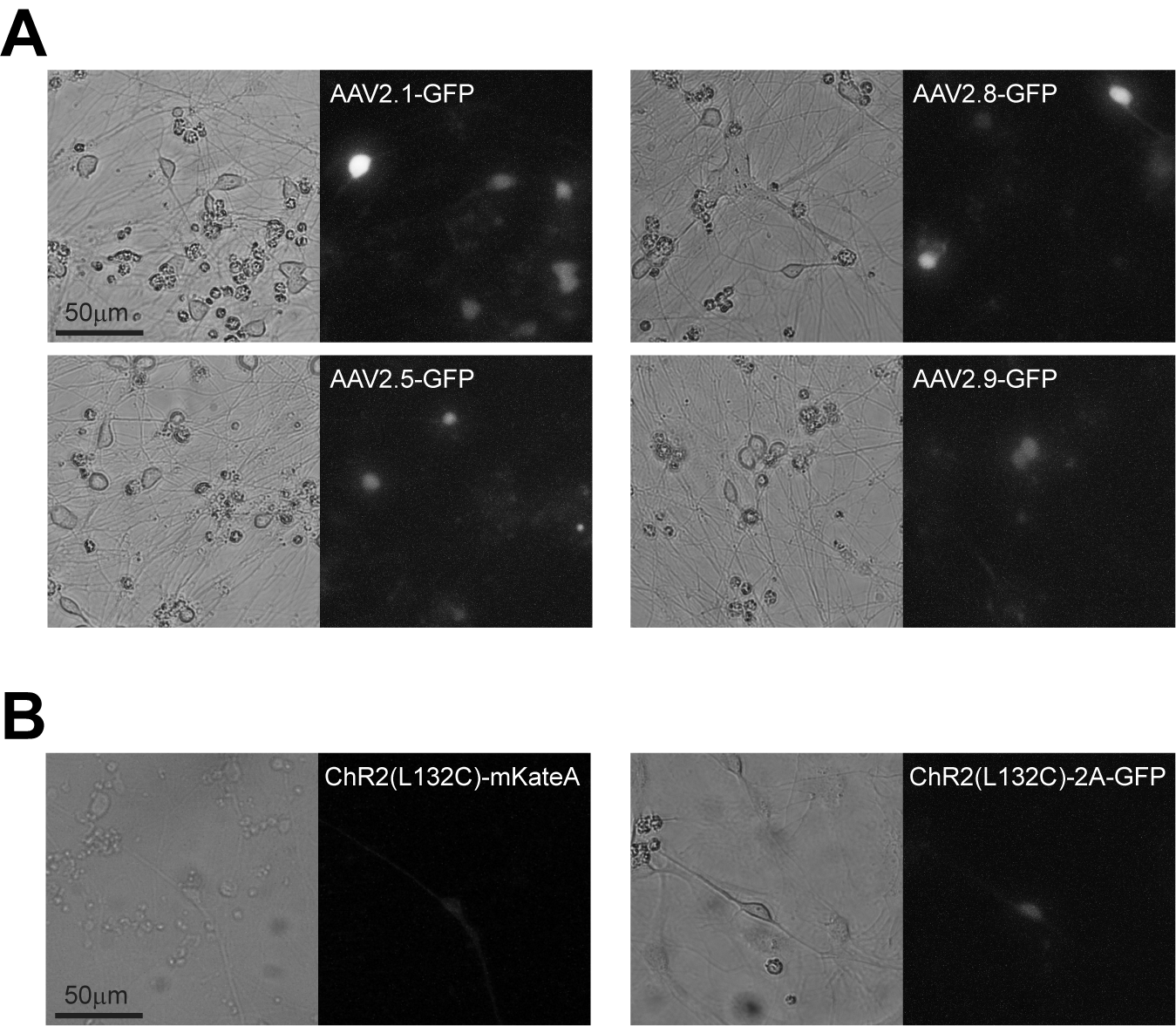

Supplement: S3 Fig — Representative pairs of microscope images of iNGNs show bright-field images on the left side and fluorescent images on the right side. (A) GFP expression using four different AAV serotypes. Images were taken from 15d iNGNs, 14 days after addition of the AAVs. (B) Two different ChR2(L132C) constructs expressed in iNGNs. The images on the left were taken from 29d iNGNs and show mKateA fluorescence 28 days after addition of AAV2.1-Synapsin-1-hChR2(L132C)-mKateA. The images on the right were taken from 15d iNGNs and show GFP fluorescence 14 days after addition of AAV2.2-CAG-ChR2(L132C)-2A-NLS-eGFP-WPRE-bGH. (TIF) [file pone.0169506.s003.tif]

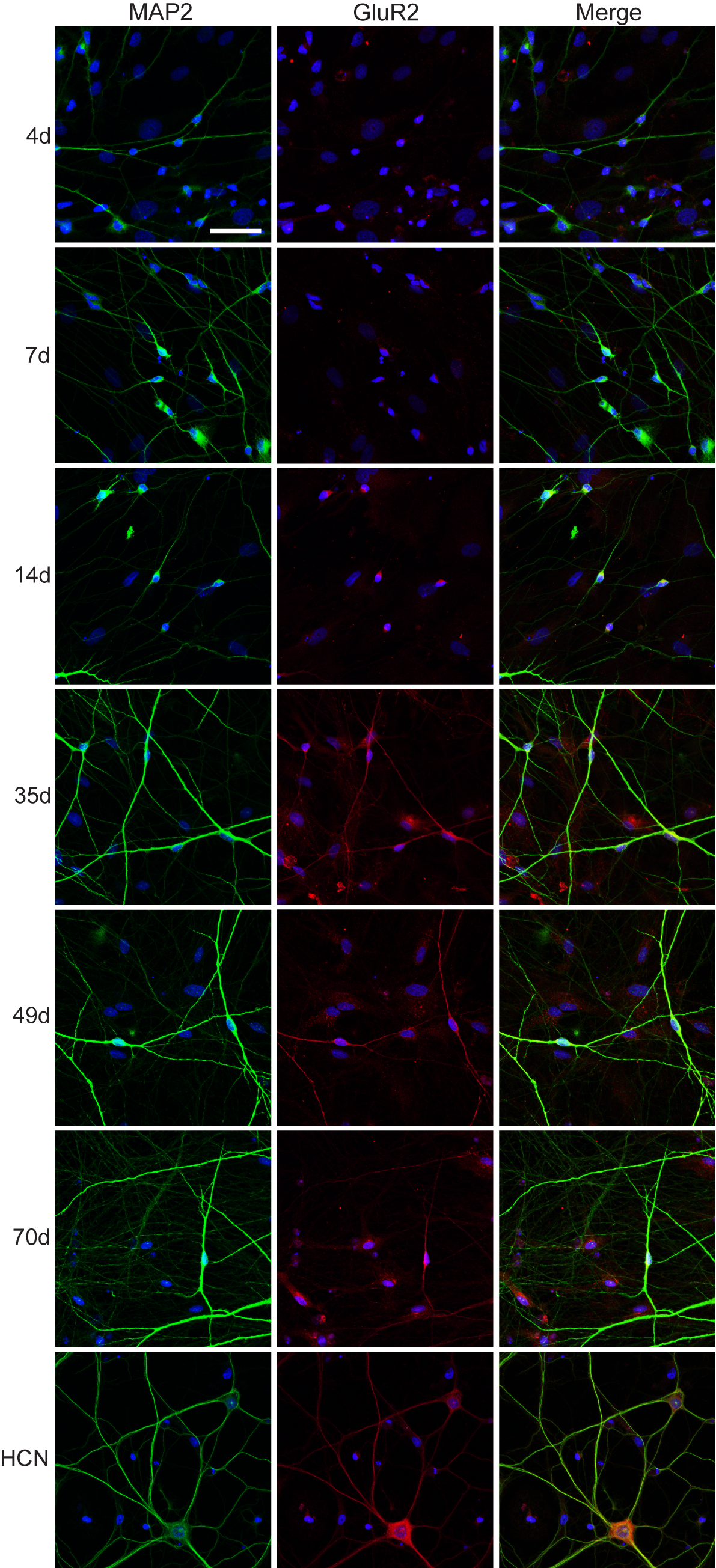

Supplement: S4 Fig — Representative z-stack confocal microscope images of 4d, 14d, 28d, 35d, 42d, and 70d iNGNs, or of 21 DIV rat hippocampal neurons (HCN). Immunocytochemistry was used to observe MAP2 (green) and GluR1 (red) expression and colocalization in iNGNs, along with the nuclear stain DAPI (blue). Ages in days (d) are labeled on the left side of the images, and the white scale bar is 50 μm long. (TIF) [file pone.0169506.s004.tif]

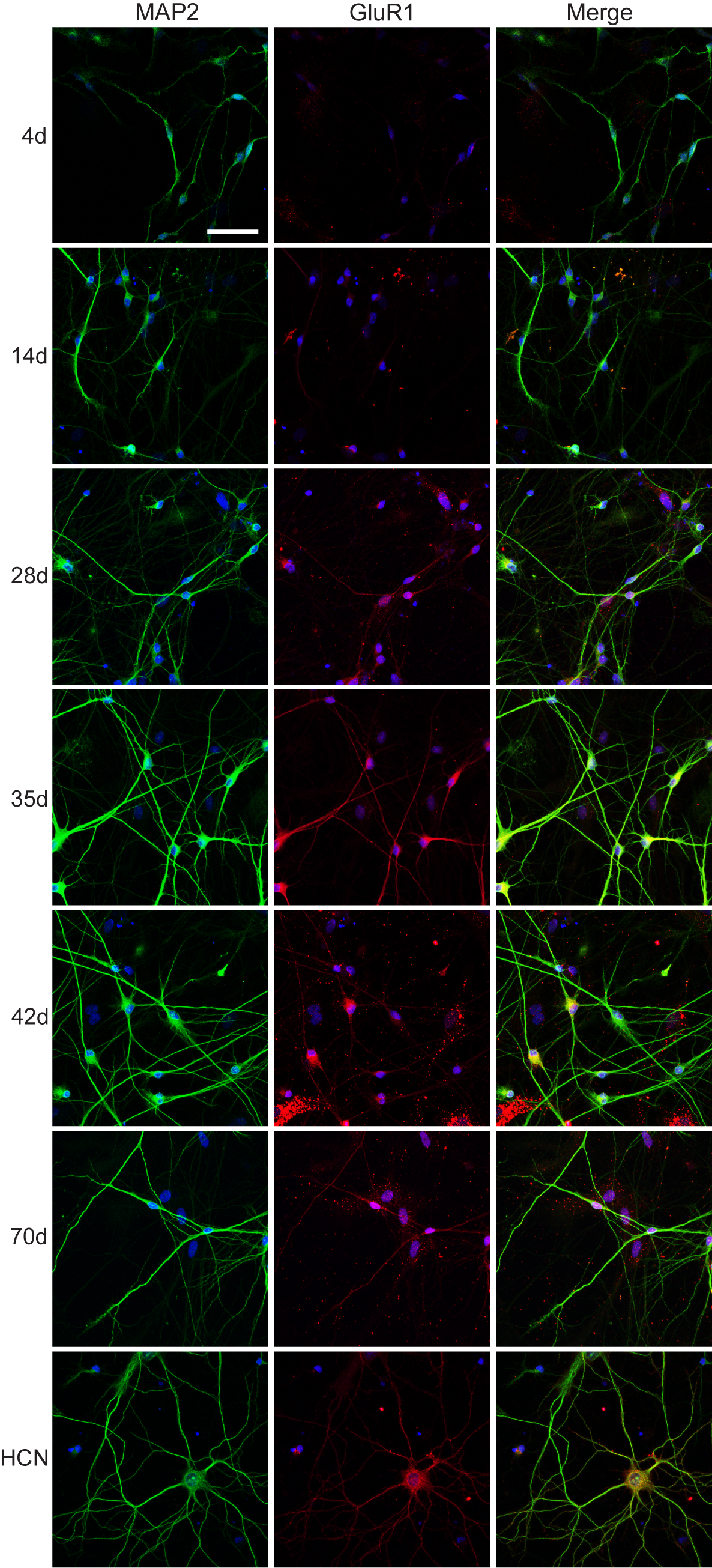

Supplement: S5 Fig — Representative z-stack confocal microscope images of 4d, 7d, 14d, 35d, 49d, and 70d iNGNs, or of 21 DIV rat hippocampal neurons (HCN). Immunocytochemistry was used to observe MAP2 (green) and GluR2 (red) expression and colocalization in iNGNs, along with the nuclear stain DAPI (blue). Ages in days (d) are labeled on the left side of the images, and the white scale bar is 50 μm long. (TIF) [file pone.0169506.s005.tif]
